# PRAME induces genomic instability in uveal melanoma

**DOI:** 10.1038/s41388-023-02887-0

**Published:** 2023-11-29

**Authors:** Stefan Kurtenbach, Margaret I. Sanchez, Jeffim Kuznetsoff, Daniel A. Rodriguez, Natalia Weich, James J. Dollar, Anthony Cruz, Sarah Kurtenbach, Matthew G. Field, Michael A. Durante, Christina Decatur, Mahsa Sorouri, Fan Lai, Gulum Yenisehirli, Bin Fang, Ramin Shiekhattar, Daniel Pelaez, Zelia M. Correa, Ramiro E. Verdun, J. William Harbour

**Affiliations:** 1 Bascom Palmer Eye Institute, University of Miami Miller School of Medicine, Miami, FL USA; 2 Sylvester Comprehensive Cancer Center, University of Miami Miller School of Medicine, Miami, FL USA; 3 Interdisciplinary Stem Cell Institute, University of Miami Miller School of Medicine, Miami, FL USA; 4 Minnesota Eye Consultants, Bloomington, MN USA; 5 Department of Ophthalmology and Simmons Comprehensive Cancer Center, University of Texas Southwestern Medical Center, Dallas, TX USA; 6 School of Life Sciences, Yunnan University, Kunming, China; 7 Proteomics and Metabolomics Core, The Moffitt Cancer Center and Research Institute, Tampa, FL USA

**Keywords:** Oncogenes, Metastasis

## Abstract

PRAME is a CUL2 ubiquitin ligase subunit that is normally expressed in the testis but becomes aberrantly overexpressed in many cancer types in association with aneuploidy and metastasis. Here, we show that PRAME is expressed predominantly in spermatogonia around the time of meiotic crossing-over in coordination with genes mediating DNA double strand break repair. Expression of PRAME in somatic cells upregulates pathways involved in meiosis, chromosome segregation and DNA repair, and it leads to increased DNA double strand breaks, telomere dysfunction and aneuploidy in neoplastic and non-neoplastic cells. This effect is mediated at least in part by ubiquitination of SMC1A and altered cohesin function. PRAME expression renders cells susceptible to inhibition of PARP1/2, suggesting increased dependence on alternative base excision repair pathways. These findings reveal a distinct oncogenic function of PRAME that can be targeted therapeutically in cancer.

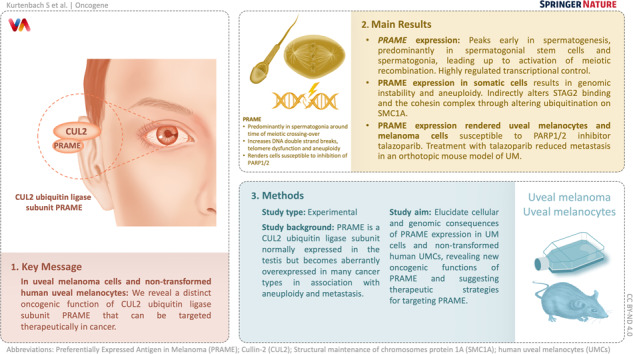

## Introduction

Preferentially Expressed Antigen in Melanoma (PRAME) was initially discovered as a melanoma antigen recognized by autologous T cells [1]. PRAME is normally expressed predominantly in the testis and becomes aberrantly expressed in a wide variety of cancer types, often associated with poor outcome [2–7]. One such cancer is uveal melanoma (UM), the most common primary malignancy of the eye, which results in metastatic disease in up to one half of patients [8]. About a quarter of UM tumors express PRAME, which is strongly associated with aneuploidy, metastasis and poor patient outcome [2, 9, 10].

Numerous functions have been attributed to PRAME, but it remains unclear how PRAME drives cancer progression. PRAME can repress retinoic acid receptor signaling and is induced by pathogen associated molecular particles (PAMPs) and gamma-interferon in leukemia cells [4, 11, 12]. PRAME can promote epithelial-to-mesenchymal transition in triple negative breast cancer [13]. PRAME functions as a substrate recognition subunit for a Cullin2-based E3 ubiquitin ligase (CUL2) complex [12, 14], and it co-localizes on chromatin with NFY and EKC/KEOPS transcription factor complexes [14, 15]. The tumor suppressor p14/ARF is one of few proteins that has been implicated as a substrate of PRAME-CUL2 [16]. Here, we elucidate the cellular and genomic consequences of PRAME expression in UM cells and non-transformed human uveal melanocytes (UMCs), revealing new oncogenic functions of PRAME and suggesting therapeutic strategies for targeting PRAME.

## Results

### *PRAME* is normally expressed in spermatogonia leading up to meiotic recombination

**Fig. 1:**
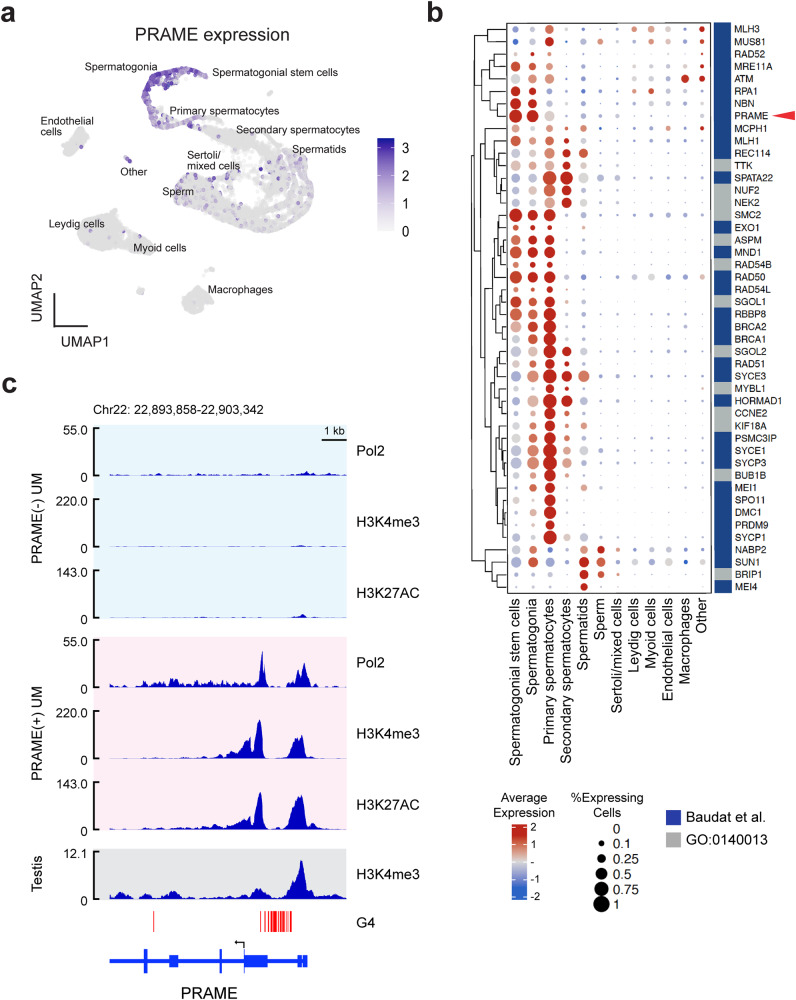
PRAME expression is tightly regulated and restricted to early spermatogenesis. **a** UMAP plot of single-cell RNA sequencing (scRNA-seq) data from human testis indicating PRAME expression. **b** Dot plot of scRNA-seq data highlighting genes involved in meiosis from Baudat et al. [18] and GO0140013. Hierarchical clustering was performed using Ward’s method. **c** ChIP-seq for RNA polymerase II (Pol2), H3K4 trimethylation (H3K4me3), and H3K27 acetylation (H3K27ac) in PRAME-negative 92.1 UM cells and PRAME-expressing Mel202 UM cells. G-quadruplex forming secondary DNA structures (G4) are marked with red vertical bars. PRAME translation start site is indicated with a black arrow.

Since *PRAME* expression is largely confined to the testis [9, 17], we analyzed single-cell RNA sequencing (sc-RNAseq) data from human testis and found that *PRAME* expression peaks early in spermatogenesis, predominantly in spermatogonial stem cells and spermatogonia, leading up to activation of meiotic recombination (Fig. 1a). The pattern of *PRAME* expression closely parallels that of essential mediators of meiotic recombination, including ATM, MRE11, RAD50, NBN, BRCA1 and BRCA2 [18, 19] (Fig. 1b).

The *PRAME* promoter region is hypermethylated and silenced in somatic cells and *PRAME*(−) cancer cells, whereas it is hypomethylated in testis and PRAME(+) cancer cells [9, 20] (Fig. 1c). Interestingly, the upstream untranslated region of the *PRAME* locus contains an unusually long stretch of approximately 36 putative G-quadruplex (G4) forming sequences (Fig. 1c), which have been associated with transcriptional regulation and cancer [21]. G4 structures are frequently formed in stem cells at promoter and enhancer regions of pluripotency genes and then lost during differentiation with silencing of these genes [21]. G4 structures regulate transcription through multiple epigenetic mechanisms; they often co-localize with the activating histone mark H3K4me3 and the histone acetyltransferase p300, and they promote DNA hypomethylation by sequestering DNMT1 [22]. Indeed, we found that UM cells expressing PRAME exhibited marked accumulation of H3K4me3 and H3K27ac surrounding the G4-forming stretch, similar to human testis, whereas these marks were absent in UM cells lacking PRAME expression (Fig. 1c). The highly regulated transcriptional control of *PRAME* and its restricted expression to meiotic cells suggest that context-inappropriate expression of *PRAME* in somatic cells has deleterious consequences and is strongly suppressed.

### PRAME activates meiotic genes and promotes chromosomal instability

**Fig. 2:**
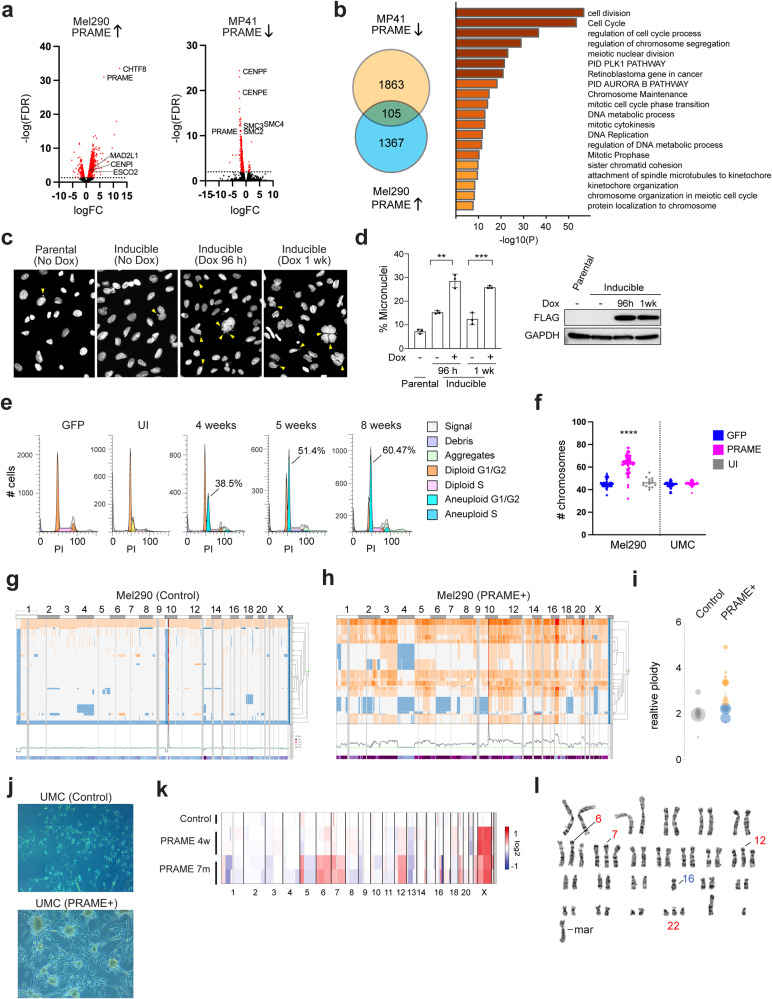
PRAME induces aneuploidy in uveal melanoma cells and non-transformed uveal melanocytes. **a** Volcano plots of RNA-seq data showing differential gene expression in Mel290 cells following enforced PRAME expression (PRAME↑) and in MP41 cells following PRAME knockout (PRAME↓). Differentially expressed genes (FDR < 0.05) are highlighted in red. **b** Venn diagram showing overlap in genes down-regulated following PRAME knockout in MP41 cells (PRAME↓), and up-regulated following PRAME expression in Mel290 cells (PRAME↑). Significantly enriched pathways containing overlapping genes are shown on the right (*p* < 0.05). **c** Representative examples of Mel290 cells (parental and PRAME-inducible) with or without doxycycline induction. Yellow arrowheads show micronuclei. **d** Summary of *n* = 3 experiments depicted in panel (**c**), showing percentage of micronuclei. Error bars represent mean ± SD. Statistical significance was determined by 2-tailed unpaired *t*-test, **p ≤ 0.01, ***p ≤ 0.001. Western blot for FLAG-PRAME expression in lysates from cells shown in the bar graph is shown on the right. **e** Flow cytometry (PI staining) showing increased numbers of aneuploid cells in Mel290 cultures following PRAME induction. Percentage of cells in the emerging aneuploid clone is indicated. **f** Chromosome numbers counted from metaphase spreads (<15 cells per group) in Mel290 UM cells and non-transformed human UMCs, following 4 weeks of PRAME expression. Statistical significance was determined by unpaired 2-tailed *t*-test with 40–70 cells per group (****p < 0.0001). **g**, **h** Single cell DNA sequencing of Mel290 cells following 4 weeks of PRAME expression. Orange indicates increased ploidy, blue decreased ploidy. **i** Relative ploidy of major clones in (**g**, **h**). **j** Phase contrast photomicrographs of control UMCs and UMCs expressing PRAME for 7 months. **k** Inferred copy number variations using single-cell RNA sequencing (scRNA-seq) in control UMCs and UMCs expressing PRAME for 4 weeks and 7 months. **l** Representative karyotype of UMCs expressing PRAME for 7 months. UI uninduced control, UMC uveal melanocytes, PI propidium iodide, Mar marker chromosome (structurally abnormal).

To explore the transcriptional impact of PRAME expression, we generated cell lines to allow for ectopic expression and knockout of PRAME (Supplementary Fig. 1). We then performed RNA-seq in (1) PRAME-negative Mel290 UM cells before and after induction of PRAME expression, and (2) PRAME-positive MP41 UM cells before and after CRISPR-mediated knockout of PRAME. The overlap of genes upregulated with PRAME expression and downregulated with PRAME knockout were enriched for pathways regulating meiosis, chromosome segregation, and DNA double strand break (DSB) repair (Fig. 2a, b). Within 4–7 days of enforced PRAME expression in the near-diploid Mel290 UM cell line, there was a significant increase in the number of micronuclei (Fig. 2c, d), which are associated with defects in mitotic chromosome segregation [23]. Within 4 weeks of PRAME expression, subclones appeared with new chromosome copy number aberrations (CNAs) demonstrated by flow cytometry and single-cell DNA sequencing (scDNA-seq) (Fig. 2e–i). In non-transformed diploid human UMCs, 7 months of enforced PRAME expression resulted in striking morphologic changes consistent with malignant transformation, including loss of contact inhibition, formation of multicellular spheroids, and aneuploidy (Fig. 2j–l). Normal UMCs cultured under the same conditions for seven months remained diploid. 15% of analyzed metaphase spreads from PRAME expressing cells harbored an abnormal chromosome 6, monosomy 16, and a marker chromosome, whereas 85% showed these abnormalities plus additional copies of chromosome 6, 7, 12 and 22 (Supplementary Table 1). These findings demonstrate that PRAME expression in somatic cells can elicit aberrant activation of transcriptional programs resulting in genomic instability and aneuploidy. Consistent with these findings, PRAME expression is associated with increased aneuploidy in multiple TCGA cancer types (Supplementary Fig. 2).

### PRAME alters cohesin complexes

**Fig. 3:**
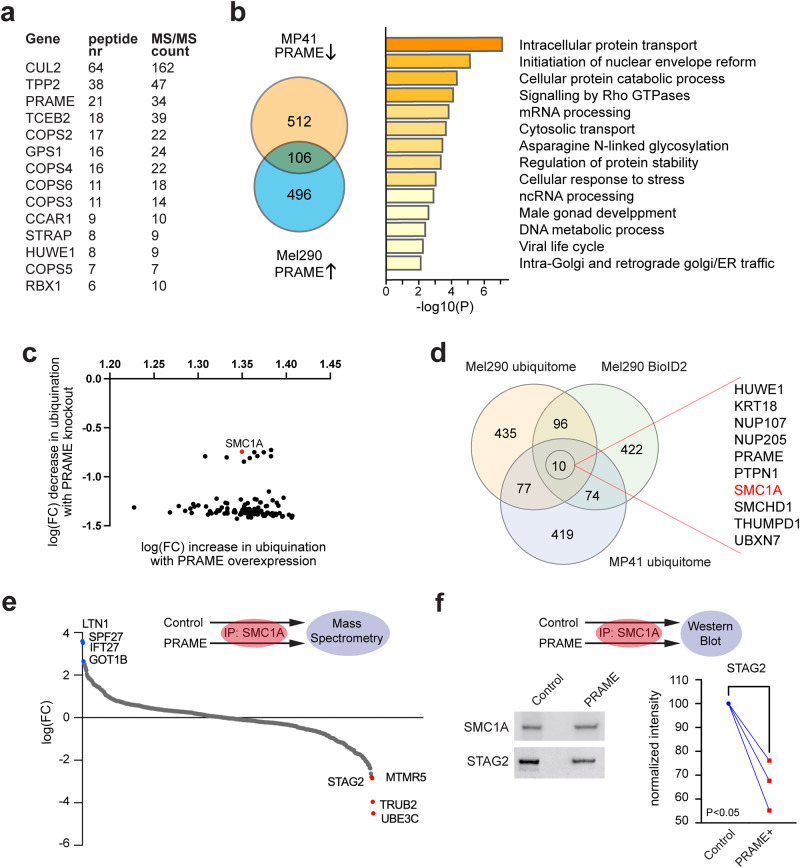
PRAME expression leads to SMC1A ubiquitination and altered cohesin composition. **a** Summary of mass spectrometry showing proteins with greatest enrichment based on number of unique peptides identified per target (peptide nr) and total number of peptides identified per target (MS/MS count) when co-immunoprecipitated with FLAG-tagged PRAME in Mel270 cells. **b** Summary of ubiquitome mass spectrometry, showing overlap of proteins with increased ubiquitination following PRAME expression in Mel290 cells (PRAME ↑), and decreased ubiquitination following PRAME knockout in MP41 cells (PRAME ↓). Significantly enriched pathways of the overlapping genes are depicted on the right (fold change > 5.0). **c** Proteins showing increased ubiquitination following PRAME expression in Mel290 cells, and decreased ubiquitination with PRAME knockout in MP41 cells. **d** Overlap of proteins enriched in ubiquitome mass spectrometry and BioID proximity ligation experiments. **e** Fold change of proteins co-immunoprecipitated with SMC1A and detected by mass spectrometry following PRAME expression in Mel290 cells. Blue dots indicate proteins with increased abundance, and red dots proteins with decreased abundance. **f** Western blots of SMC1A and STAG2 following co-IP of SMC1A in Mel290 with or without PRAME expression. Densitometric quantification of 3 replicates is shown on the right. PRAME overexpression experiments were conducted after 4 weeks of PRAME expression. FC fold change over control.

To explore how PRAME induces aneuploidy, we immunoprecipitated ectopically expressed FLAG-tagged PRAME in Mel270 UM cells and searched for PRAME-interacting proteins using mass spectrometry (Fig. 3a and Supplementary Table 2). Proteins pulled down with PRAME were enriched for components of the Cullin-RING-based E3 ubiquitin ligase (CRL) and COP9 signalosome complexes, consistent with findings in other cell types implicating PRAME as a CUL2 substrate recognition subunit [14]. To identify potential substrates of the PRAME-CUL2 complex, we probed for changes in the ubiquitinated proteome following PRAME induction in Mel290 cells and PRAME knockout in MP41 cells (Fig. 3b, c and Supplementary Table 3). Differentially ubiquitinated proteins were enriched for pathways involved in nuclear envelope reformation, male gonad development, and cellular response to stress. We then expressed BioID2-tagged PRAME in Mel290 cells to perform proximity labeling followed by pull down and mass spectrometry to screen for additional protein-protein interactions (Supplementary Table 4). The intersection of proteins enriched in these three experiments yielded a list of 10 leading candidates for PRAME-CUL2 substrates, including SMC1A and other proteins involved in chromosome maintenance (e.g., HUWE1, NUP107 and SMHD1) (Fig. 3d).

We examined how PRAME may specifically affect the function of SMC1A, a core subunit of the cohesin complex that is essential for proper chromosome segregation in dividing cells [24]. PRAME expression resulted in increased ubiquitination of SMC1A at K998 (Supplemenatry Table 3), yet its protein levels were not decreased (Supplementary Fig. 3), suggesting that PRAME-directed ubiquitination does not result in proteasomal degradation of SMC1A but rather, to altered function. As such, we hypothesized that PRAME-directed ubiquitination of SMC1A may affect its function by altering its interaction with other cohesin components. To investigate this possibility, we immunoprecipitated SMC1A and performed mass spectrometry in Mel290 cells with or without induction of PRAME (Fig. 3e and Supplementary Table 5). PRAME expression resulted in decreased interaction between SMC1A and several proteins, including STAG2, a cohesin subunit associated with centromere cohesion [25–27]. This finding was orthogonally validated in co-immunoprecipitation experiments, wherein PRAME significantly decreased the interaction between SMC1A and STAG2 (Fig. 3f). We did not observe direct interaction of PRAME to SMC1A or STAG2 by co-IP (Supplementary Fig. 4), and SMC1A localization appeared un-altered by ICC (Supplementary Fig. 5). Together, this data suggests that PRAME might indirectly alter STAG2 binding and the cohesin complex through altering ubiquitination on SMC1A.

### PRAME induces genomic instability

**Fig. 4:**
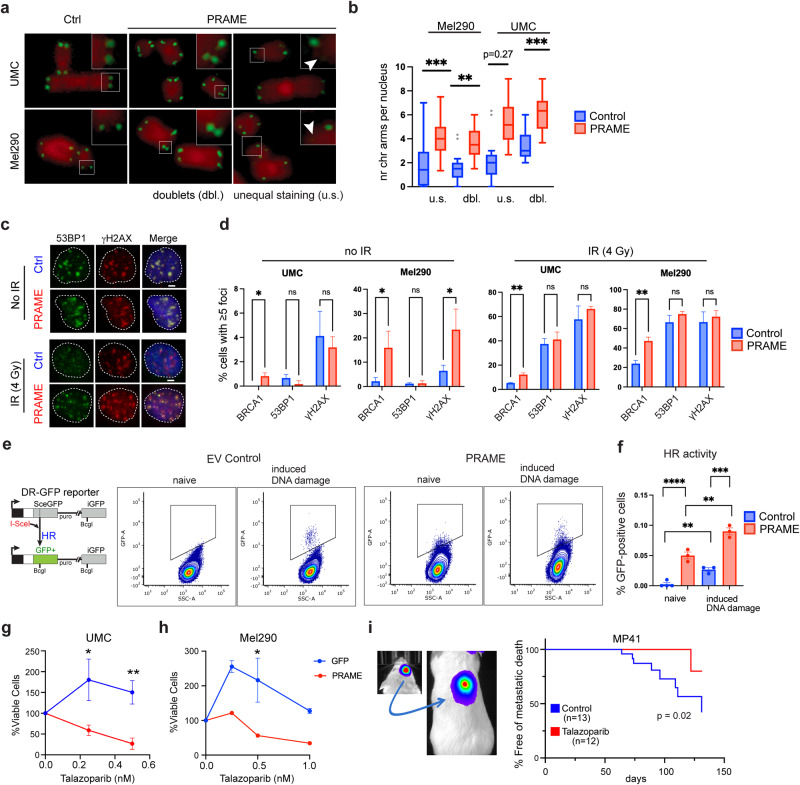
PRAME induces telomere instability and DNA double strand breaks. **a** Representative examples of unequal telomere staining (u.s.) and telomere doublets (dbl.) in Mel290 cells and non-transformed uveal melanocytes (UMC), control or expressing PRAME. **b** Quantification of chromosome arms with unequal telomere staining (u.s.) or telomere doublets (dbl.) per nucleus in Mel290 and UMC cells, control or expressing PRAME. **c** Representative examples of staining for 53BP1, gH2Ax, and BRCA1 foci, with or without 4 Gy irradiation (IR) in >150 Mel290 and UMC cells, with *n* = 3 for control (Ctrl) and expressing PRAME. **d** Quantification of staining for 53BP1, gH2AX, and BRCA1 foci in Mel290 and UMC cells, control or expressing PRAME (% cells with 5 or more foci). **e** Schematic of the DR-GFP assay, and representative examples of flow cytometry for direct repeat green fluorescent protein (DR-GFP) HR reporter assay in which cells express GFP as a result of homologous recombination (HR) of double strand DNA breaks (DSBs) before and 96 h after SceI transfection in >400,000 Mel290 cells expressing PRAME or empty vector (EV) control, with *n* ≥ 3 per group. **f** Quantification of DR-GFP HR reporter assay results from 3 independent experiments (**p < 0.01, ***p < 0.001, ****p < 0.0001). **g**, **h** PRAME expression decreases cell viability following PARP inhibition with talazoparib in UMCs and Mel290 cells expressing PRAME or GFP control (*p < 0.05, **p < 0.01). **i** Orthotopic mouse model in which MP41 UM cells with high endogenous PRAME expression are implanted in the suprachoroidal space of NSG mice and metastasize to the liver within 8–12 weeks. Kaplan-Meier survival curves demonstrate the effect of treatment with talazoparib. PRAME overexpression experiments were conducted after 4 weeks of PRAME expression.

Cohesin complexes play a key role in sister chromatid cohesion, telomere maintenance and DNA damage repair [24, 28], which are critical to maintaining genomic stability. Using fluorescence in situ hybridization (FISH) to probe telomere integrity, we found that induction of PRAME in both UMC and Mel290 cells caused an increase in telomere loss and doublet formation (Fig. 4a, b), consistent with fragile telomeres and a defect in DNA repair activity [29, 30]. Hence, we determined the activity of the two main DSB repair pathways, homologous recombination (HR) and non-homologous end-joining (NHEJ), by evaluating the accumulation of HR and NHEJ core factors BRCA1 and 53BP1, respectively in cells that were either unchallenged or treated with ionizing radiation (IR) to induce DSBs. As expected, 4 Gy of IR induced comparable levels of gH2AX staining, a marker of DSBs, in both UMC and Mel290 cells (Fig. 4c, d). Interestingly, PRAME expression increased the number of gH2AX foci in the absence of IR in Mel290 cells, indicating that PRAME alone can induce the accumulation of DSBs. Further, recruitment of BRCA1, but not 53BP1, was significantly increased by PRAME expression, with or without IR in both UMC and Mel290. Since BRCA1 recruitment is a hallmark of HR-mediated DNA damage repair, we further evaluated the effect of PRAME expression on HR in Mel290 cells using a direct repeat green fluorescent protein (DR-GFP) HR reporter assay [31]. PRAME expression resulted in a significant increase in HR activity in the presence or absence of SceI endonuclease activity (Fig. 4e, f). Since PRAME expression leads to an increase in DSBs, we hypothesized that it may also result in susceptibility to inhibitors of poly(ADP-ribose) polymerase 1/2 (PARP1/2), which are central to DNA damage repair pathways [32]. Indeed, PRAME expression rendered UMCs and Mel290 cells susceptible to the PARP1/2 inhibitor talazoparib (Fig. 4g, h). In an orthotopic mouse model of UM using MP41 cells, which express high endogenous levels of PRAME, treatment with talazoparib significantly reduced metastasis (Fig. 4i). These findings indicate that PRAME dysregulation in somatic cells results in chromosomal instability that can be targeted pharmacologically.

## Discussion

PRAME is a cancer-testis antigen that is normally expressed in the testis and is aberrantly overexpressed in many cancer types [9, 17, 33]. Here, we shed new light on the functions of PRAME and reveal how misexpression of PRAME promotes genomic instability and aneuploidy, which are hallmarks of cancer [34, 35]. We found that PRAME is normally expressed preferentially in spermatogonial stem cells and spermatogonia at meiotic crossing-over, where homologous chromosomes undergo programmed DSBs, exchange of genetic material, and repair by HR [18]. Genes coordinately expressed with *PRAME* in spermatogenesis include essential mediators of HR, including *ATM, MRE11, RAD50, RPA1, NBN, BRCA1* and *BRCA2* [18, 19]. PRAME expression in UM cells and non-transformed UMCs activated transcriptional programs involved in meiosis, DNA repair and chromosomal segregation. Further, PRAME expression led to increased DSBs, HR and telomere instability, as well as the formation of micronuclei, a hallmark of chromosomal instability associated with chromosome segregation errors and aneuploidy [23, 36]. Taken together, these findings suggest that PRAME plays a role in regulating normal meiotic recombination and causes genomic instability when mis-expressed in somatic cells, which could explain the association between PRAME expression and aneuploidy in UM and numerous other cancer types (Supplementary Fig. 1) [2, 9].

PRAME misexpression appears to induce these genomic aberrations at least in part by altering the function of cohesin complexes, which align sister chromatids during HR, telomere maintenance, and chromosome segregation [24, 28, 37, 38]. PRAME expression resulted in the ubiquitination of SMC1A, a core member of the cohesin complex [24]. This ubiquitination did not result in a reduction of SMC1A protein levels, but rather, in altering the interaction of SMC1A with STAG2, another component of the cohesin complex. STAG2 mutations are associated with DNA damage repair defects and aneuploidy in numerous cancer types [39–41]. Ubiquitination modulates cohesin function during meiosis and mitosis [42–44], and further work is needed to elucidate the role of PRAME-mediated ubiquitination in tumorigenesis.

In recent years, PRAME has drawn increasing attention as an important cancer driver and potential target of immunotherapy [33, 45, 46]. Our findings suggest additional strategies for targeting PRAME through the DNA damage repair vulnerabilities it creates. PRAME expression results in an increase in DSBs despite increased HR, suggesting an increased dependency on alternative base excision repair pathways. Accordingly, we found that PRAME expression renders cells vulnerable to inhibitors of PARP1/2, which are enzymes critical to alternative DNA repair pathways [32]. The newly described functions of PRAME in this study open new avenues for investigating its role in cancer progression and for leveraging therapeutic vulnerabilities created by its expression.

## Methods

### Cell lines

UM cell lines 92.1, Mel202, Mel270 and Mel290 were cultured in 5% CO_2_ in RPMI media supplemented with 10% tetracycline-free HI FBS, 2 mM Glutamax, and 1× Pen/Strep. MP41 UM cells were cultured in 5% CO_2_ and 5% O_2_ in DMEM/F12 media supplemented with 10% tetracycline-free HI FBS, 2 mM Glutamax, 1 mM NEAA, 0.5x ITS, and 1× Pen/Strep. Non-transformed human uveal melanocytes (UMCs) were cultured in 5% CO2 and 5% O2, in Ham’s F12 supplemented with 10% tetracycline-free HI FBS, 2 mM Glutamax, 1× Pen/Strep, 100 µM IBMX, 10 ng/ml human bFGF and rSCF, and cholera toxin.

### Single-cell RNA sequencing

Human testis scRNA-seq data was obtained from a public single-cell atlas [47]. Seurat (version 3.2.2) [48] was utilized for processing and clustering cells. Cells were filtered for a minimum of 500 features and less than 20% mitochondrial RNA content. Samples were integrated using 5000 variable features with percent mitochondrial content regressed. Uniform Manifold Approximation and Projection (UMAP) dimensional reduction was conducted based on the first 30 principal components, and cells were clustered using shared nearest neighbor (SNN) algorithm with a resolution of 0.25. Cell type annotation was assigned by cell cluster, based on expression of marker genes [47]. The clustered dot plot was generated using ComplexHeatmap (Version 2.14) [49]. The dot color encodes the average RNA expression while the dot size indicates percentage of cells in a group expressing a gene of interest. Genes were hierarchical clustering based on their average RNA expression.

### Chromatin immunoprecipitation and DNA sequencing

Chromatin immunoprecipitation (ChIP) followed by next-generation sequencing (ChIP-seq) was performed using 20 million cells per experiment. Cells were crosslinked for 7 min with 1% formaldehyde, then DNA was sonicated to an average fragment size of 200–500 base pairs using a Covaris M220 sonicator. Fragmented DNA was incubated with 10 µg of the following antibodies: Pol2 (Diagenode, C15200004), H3K27AC (Active Motif, 39133), H3K4me3 (Active Motif, 39915). Libraries were prepared using the NEBNext Ultra 2 kit (NEB, E7645S) and sequenced by the OGSR with >20 million reads per sample. Reads were filtered for quality by Trim Galore! (https://github.com/FelixKrueger/TrimGalore) and aligned to the hg38 genome with Bowtie2 [50], and visualized with SparK [51]. The location of putative G-quadruplex forming regions was determined using the G4P Calculator [52].

### Plasmids and lentiviral expression vectors

The pLV-TET-PRAME-V5 vector was created by PCR amplification of human full-length PRAME cDNA fragment (Horizon # MHS6278-202802292) and subsequent recombination into a pLV-TET-C-V5 plasmid encoding C-terminal V5. Plasmids containing FLAG-tagged PRAME and BioID2-tagged PRAME were created by substituting the C-terminal V5 tag with FLAG or FLAG-BioID2 fragments, respectively, using restriction digestion and T4 ligation. MP41s with knockout of PRAME were created using lentiviral particles encoding spCAS9 (Addgene plasmid no. 50661) and guide RNA-encoding plasmid (Addgene plasmid no. 64114). Guide RNA against PRAME 5’-GGGACAGGATACAGCACGT-3’ and 5’-CCGGCAGTTAGTTATTGAG-3’ directed the CRISPR-mediated deletion of the first exon of the PRAME gene. The pLV-TRPM1-LUC vector expressing luciferase under TRPM1 promoter was synthesized by VectorBuilder. The lentiviral plasmids were packaged into lentiviral particles by transient co-transfection into HEK293T cells with pMD2G and psPAX2 packaging plasmids using JetPrime reagent (Polyplus). The lentivirus transduced cells were selected with puromycin for 7 days, clonally selected for optimal PRAME knockout or overexpression upon induction with 1 µg/ml doxycycline. Gene knockout and overexpression was verified with western blot (Supplementary Fig. 2). Biotinylation was induced with 30 µM biotin over 5 days and confirmed by WB. The TRPM1 promoter luciferase reporter plasmid was stably integrated into cells by lentiviral transduction.

### Bulk RNA sequencing

RNA was isolated with Direct-zol RNA kit (Zymo Research, USA) according to the manufacturers’ instructions. Library preparation and sequencing was conducted at the Oncogenomics Shared Facility (OGSR) of the University of Miami Sylvester Comprehensive Cancer Center. Reads were trimmed using Trim Galore! (https://github.com/FelixKrueger/TrimGalore) and aligned to the human genome build hg38/GRCH38 using STAR [53]. Read counts were normalized and batch corrected then assessed for differences in expression between groups using EdgeR [54]. Pathway analysis was conducted with Metascape [55].

### Co-Immunoprecipitation and mass spectrometry

Cells were washed in ice-cold PBS twice, lysed in lysis buffer [20 mM Tris, 137 Mm NaCl, 2 mM sodium pyrophosphate, 1 mM sodium orthovanadate, 1 mM NaF, 2 mM EDTA, 1% Triton X-100 and 10% glycerol supplemented with Halt Protease Inhibitor Cocktail (Thermo Scientific, 87786)], scraped, collected in a 1.7 mL tube and incubated by constant rotation for 1 h at 4 °C. Lysates were centrifuged at 10.000 g for 10 min at 4 °C, supernatants were placed in a new 1.7 mL tube, precleared with 25 µL of Protein A Dynabeads (Invitrogen, 10002D) for 1 h with gentle mixing at 4 °C and separated from beads with a magnet. Pre-clear lysates were incubated with 10 µg of SMC1a antibody (Bethyl, A300-055AA), 10 µg IgG Rb antibody (Proteintech, 30000-0-AP), or FLAG M2-conjugated beads (Sigma, A2220) for IP overnight at 4 °C on a rotator. Antibody IPs were bound to Protein A Dynabeads (Invitrogen, 10002D) for 2 h at 4 °C on a rotator. The beads were washed three times with ice-cold lysis buffer, divided equally into two 1.7 mL tubes and separated from the lysate by magnets. One set was used for western blotting, the other set of divided beads was sent for mass spectrometry at either the Proteomics & Metabolomics Core Facility at the Moffitt Cancer Center (Tampa, FL) or the Wistar Institute (Philadelphia, PA).

For western blotting, one set of the divided beads was eluted with SDS-loading buffer, separated through precast polyacrylamide gel (4–15%) (Bio-Rad, 5678084) and transferred to nitrocellulose membrane via Trans-Blot Turbo System (Bio-Rad, 170–4159). Membrane was blocked with 5% BSA in 1x TBS for 1 h at room temperature (RT), followed by incubation with SMC1a antibody (Cell Signaling, 6892) diluted in 5% BSA in 0.1% Tween20 in 1x TBS (TBS-T) overnight at 4 °C. Membrane was washed with 1x TBS-T three times, incubated in anti-mouse IgG horseradish peroxidase (HRP)-linked antibody (Cell Signaling, 7076 S) in TBS-T for 1 h at RT, washed with TBS-T three times, incubated with SuperSignal West Femto Maximum Sensitivity Substrate chemiluminescence reagent (Thermo Scientific, 34096) and visualized on an Amersham Imager 680 (GE Healthcare). Images were quantified using ImageJ software. Western blot was probed using antibody against STAG2 (Santa Cruz, sc-81852). The same membrane was stripped with Restore Plus Western blot stripping buffer (Thermo Scientific, 46430) and re-probed with SMC1A antibody (Bethyl, A300-055AA) to ensure equal protein loading. Anti-rabbit HRP-linked IgG (Cell Signaling, 7074P2) was used as a secondary antibody. Experiments were repeated at least three times.

For ubiquitomic and FLAG-PRAME mass spectrometry, 300 million cells per experiment were washed in cold PBS containing Halt Protease Inhibitor Cocktail (Thermo Scientific, 87786), pelleted before flash freezing, and further processed by the Proteomics & Metabolomics Core Facility at the Moffitt Cancer Center (Tampa, FL). Following lyophilization, the dried peptide pellet was re-dissolved in IAP buffer containing 50 mM MOPS pH 7.2, 10 mM sodium phosphate and 50 mM sodium chloride. Acetyl-Lysine-containing peptides were immunoprecipitated with immobilized anti-Ubiquitin Remnant Motif (K-ε-GG) antibody. (Cell Signaling Technology #5562) After two hour incubation, the antibody beads were washed 2 times with IAP buffer, followed by 3 washes with H2O. The Ubiquitinated peptides were eluted twice with 0.15% TFA. Peptide offline fractionation was performed using bRPLC cartridge (Thermo 84868) according to manufacture protocol. 4 fractions were collected from each sample. A nanoflow ultra high performance liquid chromatograph (RSLC, Dionex, Sunnyvale, CA) coupled to an electrospray bench top orbitrap mass spectrometer (Q-Exactive plus, Thermo, San Jose, CA) was used for tandem mass spectrometry peptide sequencing experiments. The sample was first loaded onto a pre-column (2 cm × 100 µm ID packed with C18 reversed-phase resin, 5 µm, 100 Å) and washed for 8 min with aqueous 2% acetonitrile and 0.04% trifluoroacetic acid. The trapped peptides were eluted onto the analytical column, (C18, 75 µm ID × 25 cm, 2 µm, 100 Å, Dionex, Sunnyvale, CA). The 120-min gradient was programmed as: 95% solvent A (2% acetonitrile + 0.1% formic acid) for 8 min, solvent B (90% acetonitrile + 0.1% formic acid) from 5% to 38.5% in 90 min, then solvent B from 50 to 90% B in 7 min and held at 90% for 5 min, followed by solvent B from 90 to 5% in 1 min and re-equilibrate for 10 min. The flow rate on analytical column was 300 nl/min. Spray voltage was 1900v. Capillary temperature was 275 °C. S lens RF level was set at 40. Sixteen tandem mass spectra were collected in a data-dependent manner following each survey scan using 15 s exclusion for previously sampled peptide peaks. MS and MS/MS resolutions were set at 70,000 and 17,500, respectively. MaxQuant (version 1.6.14.0) was used to identify modified peptides and quantify the relative intensities. Protein database was downloaded from Uniprot in March 2020. Up to 4 missed trypsin cleavage was allowed. Carbamidomethyl cystine was set as fixed modification. Lysine ubiquitination and Methionine oxidation was set as variable modification. Both PSM and peptide FDR were set at 0.05. Match between runs feature was activated.

### Telomere staining

For metaphase analysis cells were incubated with 50 ng/ml colcemide in cell culture media for 3 h, harvested by trypsinization, incubated for 10 min at RT in 75 mM KCl, and fixed in freshly prepared methanol:glacial acetic acid (3:1 v/v). Cells were stored at 4 °C and when needed dropped onto wet slides and air-dried. For FISH analysis of the metaphases the cells were pre-treated with 0.05% w/v pepsin in 10 mM HCl during 10 min at 37 °C. After washes with 1x PBS, cells were fixed with 1% formaldehyde in 1x PBS during 10 min at RT, washed again with 1x PBS and dehydrated with ethanol series (70-90-100%, 2 min each at RT) and air-dried ON. Then, cells were denatured with hybridization solution (70% deionized formamide; 2.5% 50x Denhardt’s solution; 10 mM Tris pH 7.5; 1.5 mM MgCl2) containing Alexa488-conjugated PNA probe (Alexa488-OO-(CCCTAA)3) 2 min at 80 °C on a heat block. After 4 h incubation at RT in the dark, the samples were washed twice with wash solution (70% deionized formamide and 10 mM Tris-HCl [pH 7.2]) at RT and then twice with PBS. For DAPI staining of DNA, slides with metaphase spreads were incubated 10 min in 0.5 μg/ml 4’, 6-diamino-2-phenylindole (DAPI) (Sigma) in PBS, washed with PBS for 2 min, and mounted in SlowFade Gold antifade mounting reagent (Life Technologies). Finally, the samples were analyzed as described above. Where indicated, metaphases were spread as previously described [56]. Anaphase cells were visualized by DAPI staining of cells fixed with 2% paraformaldehyde in 1× PBS and attached to slides pre-treated with poly-lysine and analyzed as previously described [56]. A total of 50 anaphases were analyzed for each cell line per experiment.

### DNA damage foci assay

Mel290 cells and UMCs with stably integrated constitutive PRAME expression vector or empty vector were plated in 100 mm dishes in duplicates. Once cells reached approximately 70% confluency, one set was exposed to 4 Gy of ionizing radiation (IR) from CIX3 320 kV Self-Contained Cabinet Irradiator (XStrahl, Suwanee, GA) and put back inside the cell incubator for 6 h. The two sets of cells (control and irradiated) were washed with 1x PBS twice, fixed with 2% formaldehyde in PBS for 10 min at RT, washed again with PBS twice and bound to poly-L-lysine coated slides. After a wash with PBS for 5 min, cells were incubated with Blocking Solution (1 mg/ml BSA, 5% horse serum, 0.1% Triton X-100 in PBS) for 30 min at RT. Next, the cells were incubated with the primary antibodies in Blocking Solution for 1 h at RT, washed three times with PBS, and incubated with donkey anti-rabbit Alexa Fluor 488 (Invitrogen, A-21206) and donkey anti- mouse Alexa Fluor 594 (Invitrogen, A-21203) for another 30 min. Finally, cells were incubated with 0.5 μg/ml 4’, 6-diamino-2-phenylindole (DAPI) (Sigma Aldrich, D9542) in PBS for 5 min, washed with PBS for 2 min, air dried, and mounted in SlowFade Diamond antifade mounting reagent (Invitrogen, S36972). Samples were analyzed using a Leica DMI6000B microscope with LASX software (Leica). At least 500 cells were analyzed per cell line/experiment. We performed 3 independent experiments. Antibodies to the following proteins were used: 53BP1 (Novus Biologicals, NB100-304), BRCA1 (EMD Millipore, 07-434), γ-H2AX (Ser 139) clone JBW301 (EMD Millipore, 05-636) and RAD51 (Bio Academia, 70-001).

### Homologous recombination assay

Mel290-EV and Mel290-PRAME cells (1 × 10^6^ cells per sample) were transfected with pDR-GFP (Addgene plasmid # 26475) using jetPRIME DNA and/or siRNA transfection reagent (Polyplus) and selected for puromycin resistance (2 μg/ml). Upon 60% confluence, the cells were transfected with a plasmid expressing the restriction enzyme I-SceI (pCBASce1) (Addgene plasmid # 26477) using jetPRIME DNA and/or siRNA transfection reagent (Polyplus). This restriction enzyme cuts the reporter plasmid and when repaired by HR, GFP is expressed [31]. Ninety-six hours after I-SceI transfection, cells were harvested, centrifuged at 300 g for 5 min at 4 °C, washed with cold 1× PBS and resuspended in cold 1× PBS. Flow cytometry and data analysis were performed by the University of Miami Sylvester Comprehensive Cancer Center Flow Cytometry Shared Resource (FCSR).

### PARP1/2 inhibition experiments

Cells were seeded into 24-well plates, three wells per cell per treatment. After 24 h, cells were treated with 0, 0.25 or 0.5 nM talazoparib (APExBIO Technology, A4153) for 5 days. Untreated cells were used as control. Cell survival was measured with trypan blue viability assay described at TC20 automated cell counter manual (Bio-Rad). The viability of the cells was normalized to control untreated group. Each experiment was performed in triplicate and repeated three times.

For in vivo mouse experiments, MP41 cells expressing luciferase were harvested, washed with 1× PBS, counted, and resuspended in PBS at a concentration of 50,000 cells/µL in a sterile laminar flow hood. Cells were maintained on ice until injection. MP41 cells (100,000 cells in 2 µL) were injected into the choroidal space of the right eye in 22 NOD.Cg-Prkdcscid Il2rgtm1Wjl/SzJ (NSG) mice using a 33-gauge needle (Hamilton, 7803-05) and a 10 µL gastight microsyringe model 1701 (Hamilton, 7653-01) under a dissecting microscope (Olympus SZX-ST). Ketamine (75 mg/kg) and xylazine (15 mg/kg) were used for anesthesia. After 2 weeks, in vivo bioluminescence imaging was performed using the In Vivo Imaging System (IVIS) Spectrum (PerkinElmer, US). Mice were then divided with equal numbers of males and females into vehicle- and talazoparib-treatment groups. Talazoparib was dissolved in 10% n,n-dimethylacetamide (DMAc) (Sigma) in 6% solutol (ChemScene) in saline (Aspen) and administered intraperitoneally (IP) every other day at 0.7 mg/kg for 60 days starting 15 days after suprachoroidal injections. Vehicle consisting of the same solution lacking talazoparib was given on the same schedule. Four weeks after suprachoroidal injections, mice were anesthetized with ketamine/xylazine, and the right eyes were enucleated to prevent local discomfort from tumor growth in the eye. A toe-pinch was performed to confirm appropriate level of anesthesia, after which the eye and lid was cleaned using aseptic techniques. Two drops of proparacaine hydrochloride ophthalmic solution 0.5% (Bausch Lomb) were instilled in the right eye before surgery as a local anesthetic, and the left eye was lubricated (Optixcare) to prevent ulceration. Next, proptosis was gently induced in the right eye using an open forceps until the globe was anterior to the lids and the optic nerve was accessible. The optic nerve and surrounding blood vessels were clamped for 5 min with a hemostat for hemostasis, and the eye was detached using surgical scissors. Enucleated eyes were placed in 10% formalin. The right eyelids were sutured together, and erythromycin ophthalmic ointment (Bausch Lomb) was applied to prevent infection. Following the surgery, mice were given one subcutaneous injection of Meloxicam SR (2 mg/kg, ZooPharm) and did not require additional analgesia based on their behavior and activity level. All mice were monitored closely and recovered fully without complications. Five months after suprachoroidal injections, mice were euthanized and necropsies were performed. Liver, lungs, kidneys, and spleen were collected, placed in 10% formalin, and sent for histopathological analysis. All experimental animal protocols used in this study were approved by the Institutional Animal Care and Use Committee at University of Miami (protocol #15–197). All activities were performed in compliance with federal, state, and institutional regulations.

### Micronuclei staining

Mel290 cells with inducible FLAG-tagged PRAME expression construct (TET-FLAG-PRAME) were grown on #1.5 coverslips, and half of replicates were treated with 1 μg/mL doxycycline for the indicated amounts of time. Cells were fixed in 4% paraformaldehyde in 1X PBS for 20 min and washed three times with 1X PBS (5 min/wash). Cells were permeabilized with 0.1% Triton X-100 in 1X PBS for 10 min and washed in 1X PBS. Coverslips were mounted using ProLong Diamond Antifade Mountant with DAPI, and cells were imaged using a Zeiss LSM 700 confocal microscope. At least 200 cells were counted for each condition and the experiments were repeated in triplicate.

### Calculation of fraction genome altered

Bulk RNA sequencing expression and fraction genome altered data from the TCGA cohort was collected for each patient across 32 cancers via the Cancer Genomics Data Server R package (cgdsr Version 1.3). For each cancer, we fitted a LOESS model of PRAME expression and identified the inflection point before expression became aberrantly high, as previously described [2, 9]. This inflection point provided the PRAME expression threshold for “positive” PRAME “positive Once samples were labeled as PRAME “HIGH” or “LOW”, we conducted a one-way Wilcox test to identify significant association (*p*-value < 0.05) of PRAME “HIGH” samples with increased fraction genome altered (FGA).

### Single-cell DNA sequencing

Single-cell DNA sequencing was performed using the Chromium platform (10X Genomics). Single-cell suspensions were counted using both the Cellometer K2 Fluorescent Viability Cell Counter and hemocytometer, and cell counts were adjusted to 1,000 cells/μl. Samples were run using the Chromium Single Cell DNA Library & Gel Bead Kit with a target capture of 500 cells. Samples were processed on Chromium Single Cell C and D Chips (10X Genomics) according to the manufacturer’s protocol and subsequently run on a thermocycler. Single-cell genomic DNA libraries were sequenced on the NextSeq 500 sequencer using 300-cycle high-output flow cells.

### Single-cell CNV analysis

Raw BCL files for the DNA sequencing data were processed using Cellranger DNA (version 1.0.0). The “mkfastq” command was used to generate FASTQ files and the “cnv” command was used to generate CNV data aligned to the 10X Genomics GRCh37 build 87 genome (version 1.0.0). Results were visualized in the Loupe scDNA Browser (version 1.0.0).

### Supplementary information

Supplementary Figure 1

Supplementary Figure 2

Supplementary Figure 3

Supplementary Figure 4

Supplementary Figure 5

Supplementary Figure Legends

Supplementary Table 1

Supplementary Table 2

Supplementary Table 3

Supplementary Table 4

Supplementary Table 5

## Data Availability

NGS sequencing data will be publicly accessible at GEO GSE231396, and GSE230484.
