# Supplementary figures and images for: PRAME induces genomic instability in uveal melanoma
Source: Oncogene. 2023 Nov 29;43(8):555–65. doi: 10.1038/s41388-023-02887-0 (PMC10873199; doi:10.1038/s41388-023-02887-0)

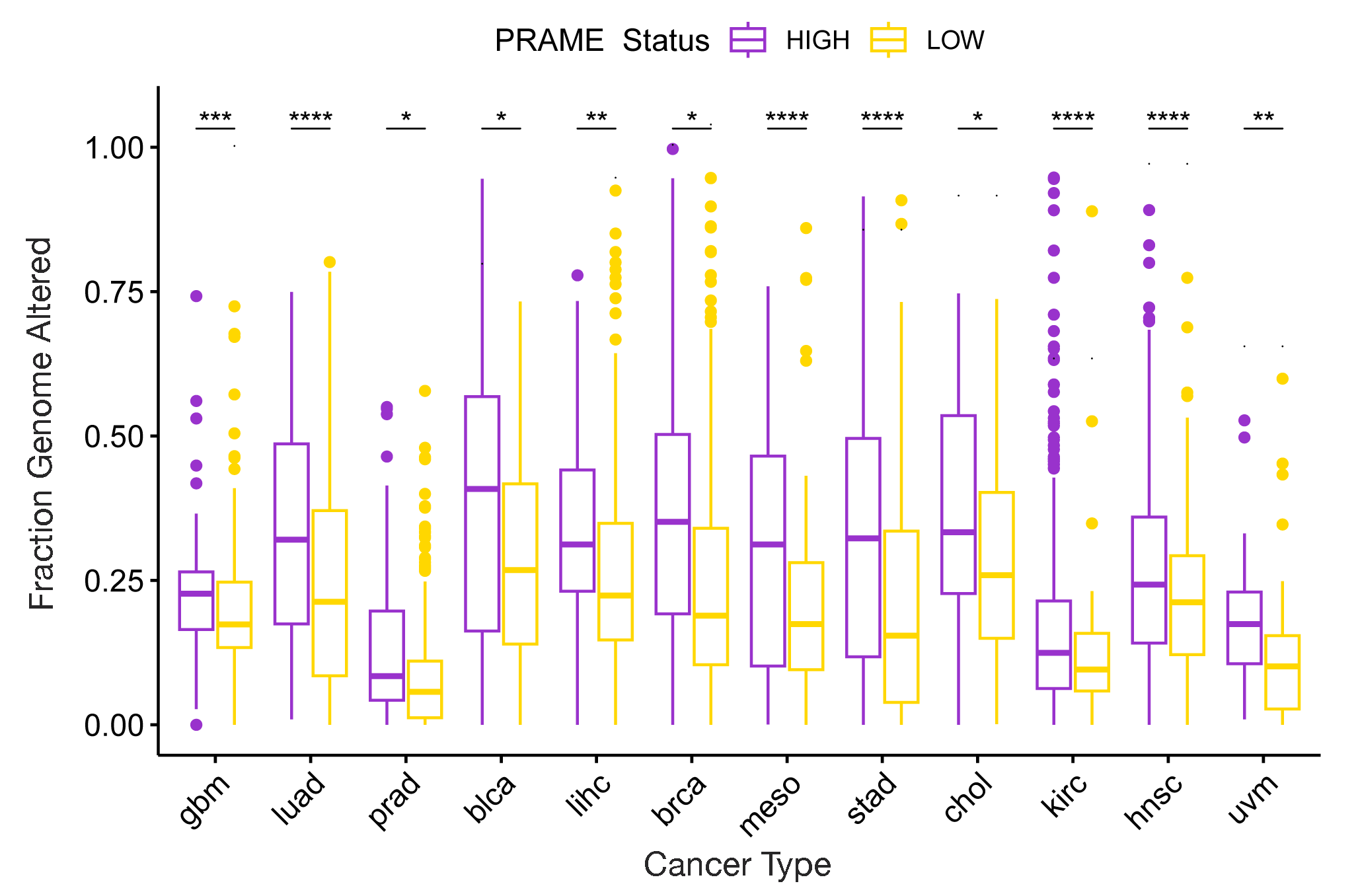

Supplement: Supplementary file 1 — Supplementary Figure 1 [file 41388_2023_2887_MOESM1_ESM.tif]

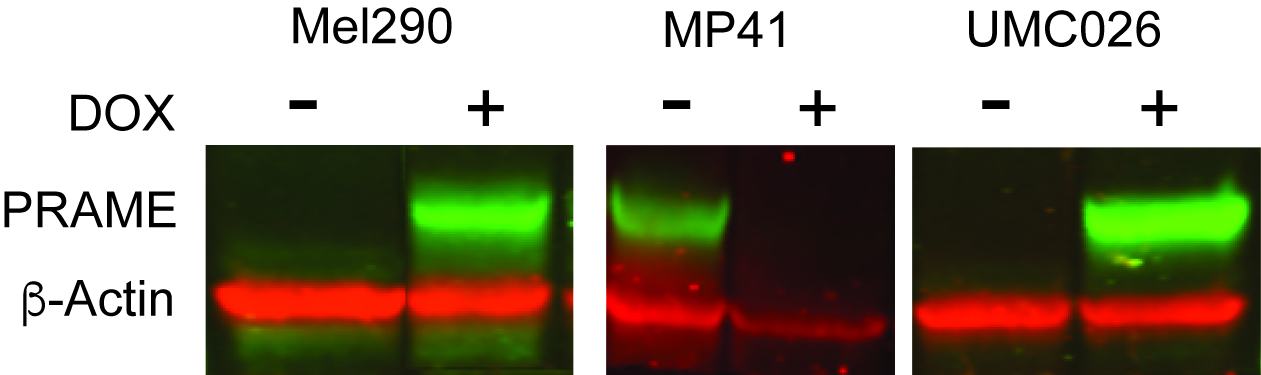

Supplement: Supplementary file 2 — Supplementary Figure 2 [file 41388_2023_2887_MOESM2_ESM.tif]

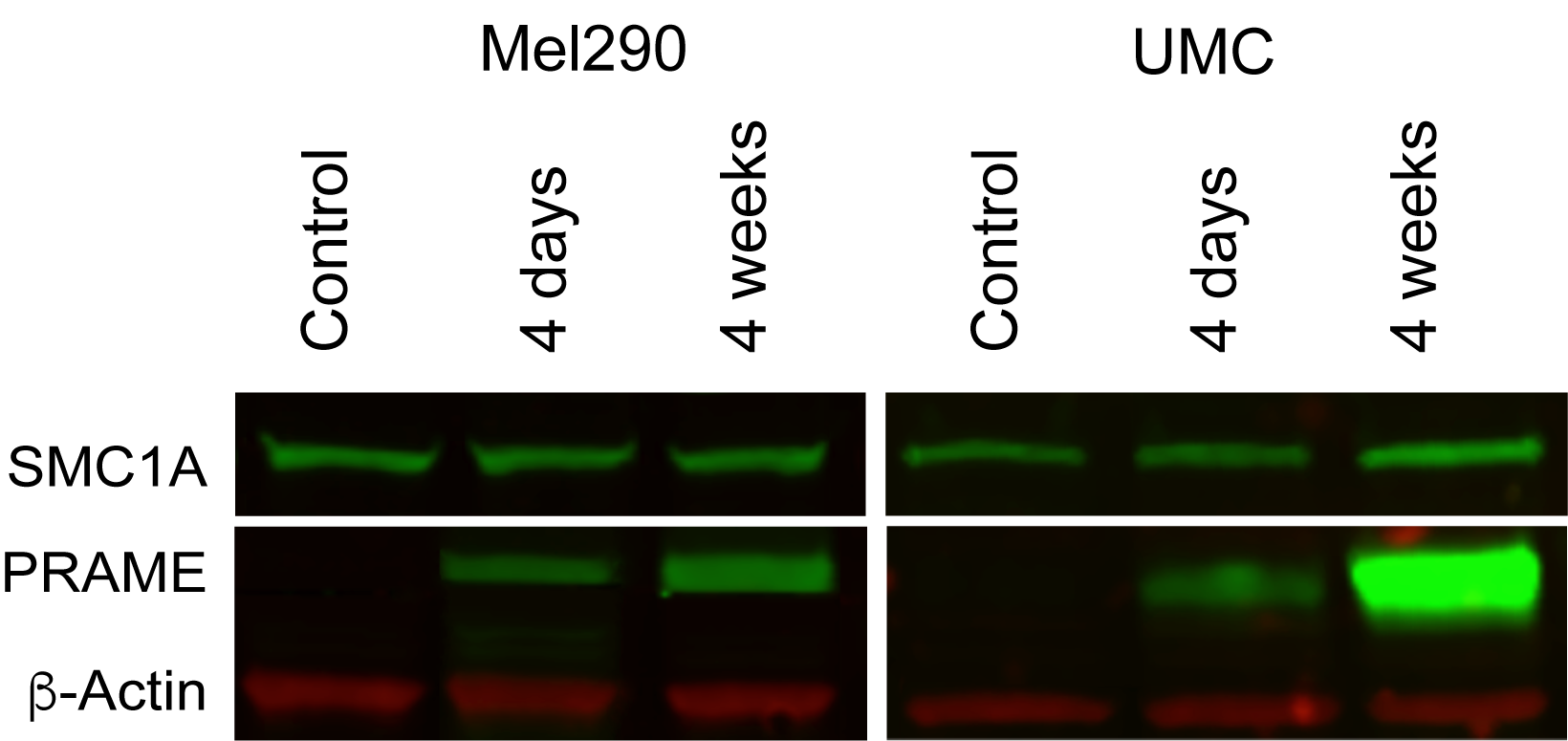

Supplement: Supplementary file 3 — Supplementary Figure 3 [file 41388_2023_2887_MOESM3_ESM.tif]

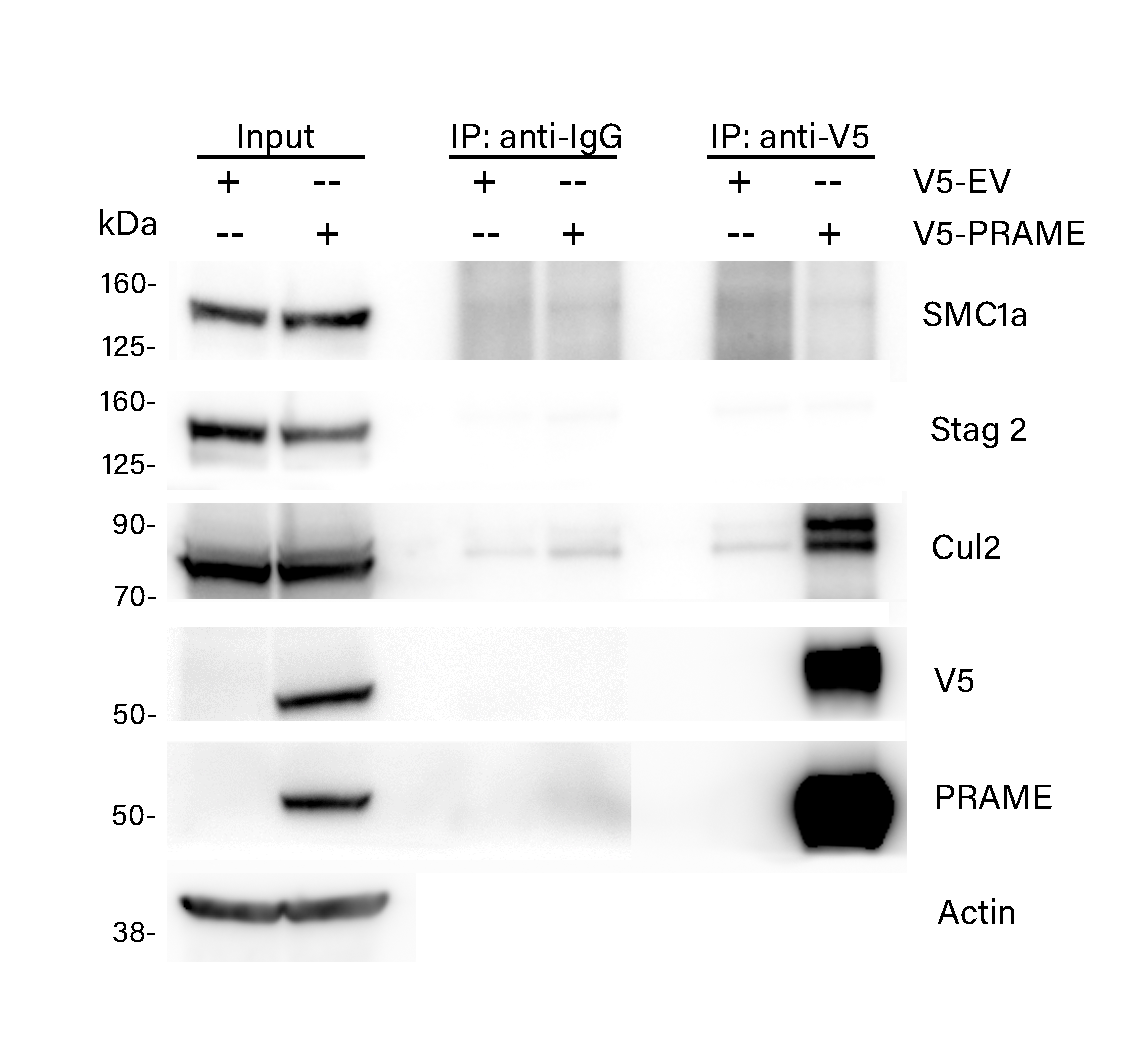

Supplement: Supplementary file 4 — Supplementary Figure 4 [file 41388_2023_2887_MOESM4_ESM.tif]

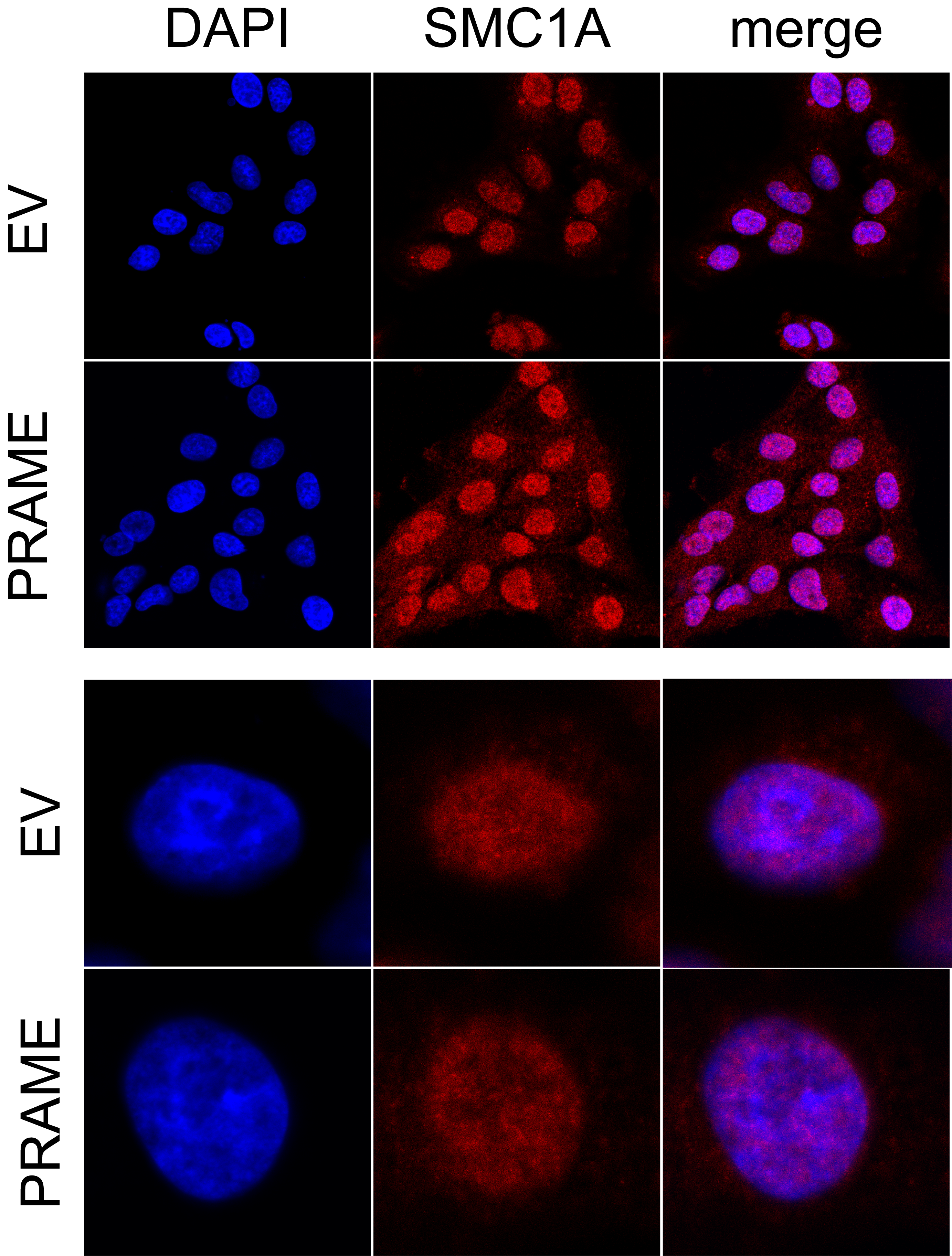

Supplement: Supplementary file 5 — Supplementary Figure 5 [file 41388_2023_2887_MOESM5_ESM.tif]
