# Supplementary material for: PRAME induces genomic instability in uveal melanoma
Source: Oncogene. 2023 Nov 29;43(8):555–65. doi: 10.1038/s41388-023-02887-0 (PMC10873199; doi:10.1038/s41388-023-02887-0)
Supplement: Supplementary file 6 — Supplementary Figure Legends [file 41388_2023_2887_MOESM6_ESM.docx]

**Supplementary Figure 1.** Western blot to analyze PRAME and β-actin protein levels before and after 1 week of doxycycline (DOX) treatment in Mel290 cells with inducible PRAME expression construct, MP41 cells with inducible shRNA construct targeting PRAME, and uveal melanocytes (UMC) with inducible PRAME expression construct

**Supplementary Figure 2.** Pan-Cancer analysis of fraction genome altered in association with increased PRAME expression in 12 cancer types from the Cancer Genome Atlas (TCGA). GMB, glioblastoma multiforme; LUAD, lung adenocarcinoma; PRAD, prostate adenocarcinoma, BLCA, bladder urothelial carcinoma, LIHC, liver hepatocellular carcinoma; BRCA, breast invasive carcinoma; MESO, mesothelioma; STAD, stomach adenocarcinoma; CHOL, cholangiocarcinoma; KIRC, kidney renal clear cell carcinoma; HNSC, head and neck squamous cell carcinoma, and uveal melanoma (UVM). Color indicates the PRAME expression status: purple, high expression; yellow, low expression. Significance: * p<0.05, **p<0.01, ***p<0.001, ****p<0.0001.

**Supplementary Figure 3.** Western blot to analyze SMC1A, PRAME, and β-actin protein levels. Depicted are the blots for Mel290 and human uveal melanocytes (UMC) following 4 days and 4 weeks of enforced PRAME expression.

**Supplementary Figure 4.** Co-immunoprecipitation of V5-tagged PRAME in V5-PRAME and V5-EV (Empty Vector) cells. We did not find direct Stag2 binding to PRAME. Cul2 is a known to be a member of the PRAME-CUL2 multi-protein complex and serves as a positive control for the IP.

**Supplementary Figure 5. IHC for SMC1A in Mel290 cells expressing PRAME.** SMC1A is located in the nucleus, with no apparent changes between PRAME and EV (Empty Vector) expressing cells. Nuclei are stained in blue with DAPI.
